# In vivo and in vitro analysis in a rat model using zoledronate and alendronate medication: microbiological and scanning electron microscopy findings on peri-implant rat tissue

**DOI:** 10.1186/s12903-021-02031-y

**Published:** 2021-12-31

**Authors:** Kristian Kniha, Eva Miriam Buhl, Stephan Christian Möhlhenrich, Anna Bock, Frank Hölzle, Elmar Hellwig, Ali Al-Ahmad, Ali Modabber

**Affiliations:** 1 Department of Oral and Cranio-Maxillofacial Surgery, University Hospital RWTH Aachen, Pauwelsstraße 30, 52074 Aachen, Germany; 2 Institute of Pathology, Electron Microscopy Facility, University Hospital Aachen, Aachen, Germany; 3 Department of Orthodontics, University of Witten/Herdecke, Alfred-Herrhausen Str. 45, 58455 Witten, Germany; 4 Department of Operative Dentistry and Periodontology, Faculty of Medicine, Albert-Ludwigs-University, 79106 Freiburg, Germany

**Keywords:** Dental implant, Zirconia, Titanium, SEM, Bacteria, Bisphosphonate

## Abstract

**Background:**

The aim of the present study was to assess the development of bacterial deposits and morphological parameters around dental zirconia and titanium implants compared with natural teeth during systemic bisphosphonate medication.

**Materials and methods:**

Fifty-four rats were randomly allocated into one control group and two experimental groups (drug application of zoledronic and alendronic acid), with 18 animals in each group. After 4 weeks of drug delivery, either a zirconia or a titanium implant was immediately inserted. Microbiological analysis conducted 1 week, 8 weeks, and 12 weeks after surgery included total bacterial count and composition measurements. Samples were analyzed in a scanning electron microscope (SEM) equipped with energy-dispersive X-ray spectroscopy (EDX). Bone cell morphology was analyzed by transmission electron microscopy (TEM).

**Results:**

One week after surgery, titanium and zirconia implants of the alendronic acid and control group showed a significantly higher bacterial count when compared to natural teeth in rats with zoledronic acid administration (*p* < 0.01). Less significant differences were recorded after 3 months, at which time no inter-material differences were evaluated (*p* > 0.05). I n the control group, TEM analysis showed that the osteoblasts had a strongly developed endoplasmic reticulum. In contrast, the endoplasmic reticulum of the osteoblasts in drug-treated animals was significantly less developed, indicating less activity.

**Conclusions:**

Within the limits of this study, neither implant material was superior to the other at 3-month follow-up. With regard to the treatment and complications of patients with bisphosphonates, the implant material should not be an influencing factor. Bisphosphonates can be used in the rat model to reduce not only the activity of osteoclasts but also osteoblasts of the peri-implant bone.

## Introduction

Bisphosphonates were first produced at the end of the eighteenth century. Their affinity for hydroxyapatite crystal surface led Procter and Gamble to evaluate these medicaments in the field of medicine [1]. The first bisphosphonate, etidronate disodium, was used in 1968 to treat a young patient with myositis ossificans progressive [2]. Zoledronic acid (relative potency > 10,000) has been approved as Zometa® since 2003 for the treatment of tumor-induced hypercalcemia and for the prevention of skeletal complications in patients with advanced tumor diseases extending to the skeleton [3]. In addition to suppressing bone resorption, clinical studies have demonstrated various anti-tumor activities that may contribute to the overall effect. Inhibition of resorption reduces the susceptibility of bone marrow to tumor cell growth, and anti-angiogenic and analogous effects have been observed [4]. Zometa is usually administered as an intravenous infusion every 3 to 4 weeks. Zoledronic acid has been approved as Aclasta® since 2005 for the treatment of osteodystrophia deformans (Paget's disease) and since 2007 for the treatment of postmenopausal osteoporosis in women [5]. Bisphosphonates are now established as an important class of drugs for the treatment of many bone diseases [6]. Alendronic acid (Fosamax®), which was first approved on December 9, 2008 and has a relative potency of 100–1000, also originates from the group of bisphosphonates and has a similar effect to zoledronic acid, but in a 20-fold lower dose [5]. In the postmenopause, bone density was increased by 3–7% after 3 years of administration, and the frequency of vertebral fractures was reduced from 6 to 3%. Oral bioavailability is 0.6%, but the terminal half-life of the release from the bone is over 10 years [7]. Further research on bisphosphonates led to the treatment of osteoporosis, Paget's disease of the bone, hypercalcemia of malignancy, and metastatic bone disease [2]. In general, bisphosphonates can be classified into two categories according to their mechanisms (nitrogen-containing and non–nitrogen-containing BPs) and each bisphosphonate behaves differently in terms of mineral binding and cellular effects [8]. The overall pharmacological effects of bisphosphonates on bone appear to depend upon their affinity for bone mineral and their inhibitory effects on osteoclasts. These effects lead to an increased failure rate with regard to implants, but also to jaw necrosis following any surgical intervention [9]. The rationality of the study was to compare different implant materials with these medications. The authors assumed that in the event of a wound healing disorder or peri-implantitis in the case of bisphosphonate administration, the bacterial accumulation and thus the number of bacteria increases. With regard to the number of bacteria, studies have shown that already at the stage of peri-implant mucositis the total number of bacteria increased [10, 11]. Thus, the total number of bacteria can be used as a marker for the degree of the individual inflammation. In general, the mechanism leading to bisphosphonate-associated ONJ, and thus whether the oral microbiome is causative, is still unclear [12]. Nevertheless, Holzinger et al. showed that the insertion of dental implants during or after bisphosphonate treatment accelerated the development of ONJ [13]. There are also numerous case series and retrospective studies of bisphosphonate-associated osteonecrosis of the jaw after implantation [13–17]. The relative effects of these properties display differences among individual bisphosphonates and lead to individual clinical behavior and effectiveness [18]. Furthermore, individual periopathogens seem to play an important role in the development of peri-implantitis. Al-Ahmad et al. showed that a shift in the healthy subgingival microbiota was observed in peri-implantitis-associated biofilm [19, 20]. Basic studies on various implant materials and with bisphosphonate administration are rare and therefore it is still unclear whether there are material-related differences in peri-implant hard and soft tissue [21–23]. Nevertheless, clinical studies of zirconia implants with healthy patients already showed positive outcome [24–30].

This study primarily aimed to evaluate the effect of the implant material, either titanium or zirconia, under systemic bisphosphonate medication on the peri-implant composition and development of bacterial deposits. In the process, different types of bisphosphonates were investigated. We also investigated the effect of systemic bisphosphonate medication on osteocytes, osteoblasts and osteoclasts of the jaw bone.

## Materials and methods

### Experimental protocol

At the beginning of the study, 54 adult male Sprague–Dawley rats, each weighing 250 g and aged 7 weeks (Janvier Labs, Le Genest-Saint-Isle, France) were included. One examiner performed the individual assessments of the study. This investigation is related to the microbiological and scanning electron microscopy (SEM) results of the study, which was carried out in accordance with the guidelines of the European Parliament and of the Council on the protection of animals used for scientific purposes, ARRIVE (Animal Research: Reporting of In Vivo Experiments) and Directive 2010/63/EU. The study protocol received ethical approved from the appropriate local authority (Landesamt für Natur und Verbraucherschutz, Recklinghausen, Germany; Ref. 2018A314).

Two experimental groups and one control group with 18 animals in each group were randomly divided as follows: zoledronic acid (Group 1), alendronic acid (Group 2), and control without any medication (Group 3). Systemic medication with antiresorptive drugs was started 4 weeks before implantation and administered for a period of 4 months. The drugs were diluted with physiologic phosphate-buffered saline before administration. Rats in Group 1 received a dose of 0.04 mg/kg body weight zoledronic acid (Mylan dura GmbH, Darmstadt, Germany) intravenously in the tail vein once per week [31]. A total of 0.2 mg/kg body weight alendronic acid (alendronate sodium trihydrate, Sigma Aldrich GmbH, Munich, Germany) was administered subcutaneously 5 times a week to rats in Group 2 [32]. The rats were provided with food and water ad libitum, with only soft soaked food supplied after implantation until the end of the investigation.

### Implant placement

**Fig. 1:**
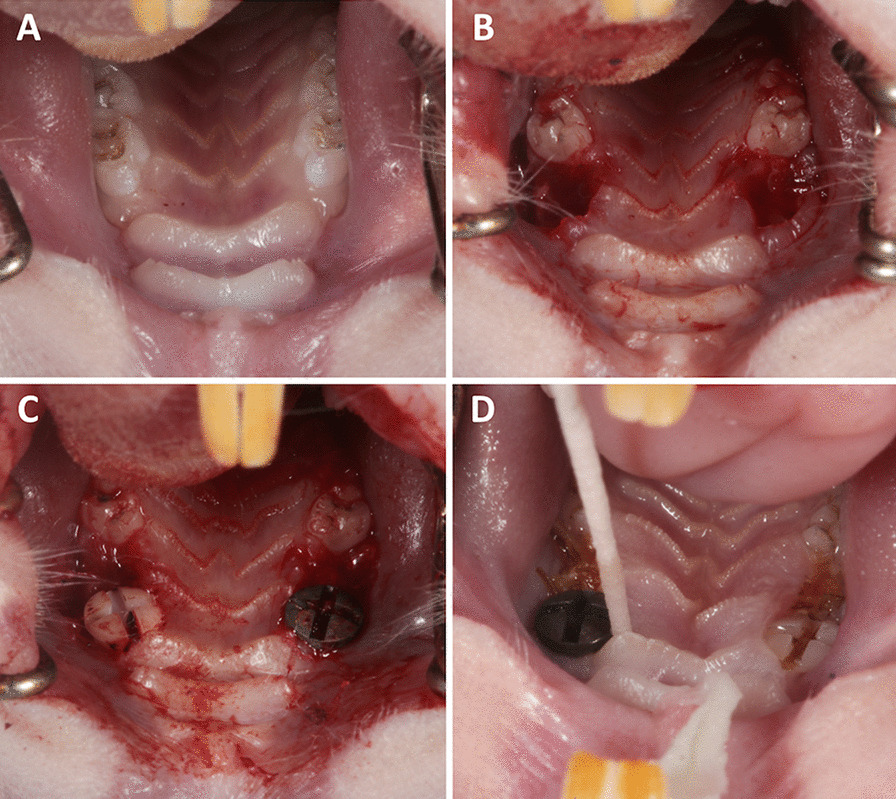
**A** Initial situation in the upper jaw of rats before surgery. **B** The first molars on both sides of the upper jaw were removed. **C** On each site, either a zirconia or a titanium implant was immediately inserted at a site randomly determined. **D** The deepest probing pocket depth around each unit (implant or tooth) was sampled for 30 s with sterile paper points

After 4 weeks of drug delivery, surgery was performed. A total of 54 microrough titanium and 54 zirconia implants with a polished shoulder (length 4 mm and diameter 2 mm) were custom-made by the Straumann Company by the same process used for commercially available implants (Institute Straumann AG, Basel, Switzerland). Sa roughness values for zirconia implants were 0.63 μm and for titanium implants 1.5 μm. The rats received an intraperitoneal anesthetic cocktail consisting of 90 mg/kg body weight ketamine (Medistar GmbH, Ascheberg, Germany) and 0.2 mg/kg body weight medetomidine hydrochloride (Domitor, Bayer Austria, Vienna, Austria). Subsequently, after extraction of the first molar of the upper jaw on each site, in a split-mouth study design either a zirconia or a titanium implant was immediately inserted in a site randomly determined (Fig. 1A–C). The implant site was prepared with a pilot drill with 2.2 mm diameter and the insertion was performed according to the manufacturer’s protocol with a transgingival healing process (Institute Straumann AG, Basel, Switzerland). At the end of the surgery, the antidote atipamezole hydrochloride (Orion Pharma, Espoo, Finland), at a dose of 0.8 mg/kg body weight, was administered subcutaneously. In the first 3 days postoperatively, the animals were treated once a day with carprofen 4 mg/kg subcutaneously (Rimadyl, Zoetis GmbH, Berlin, Germany), according to a score sheet.

### Subgingival/submucosal plaque sampling and DNA extraction

Plaque sampling was conducted 1 week, 8 weeks, and 12 weeks after surgery. For probing analysis, both implants and one randomly selected molar of the adjacent teeth (second molar of the upper rat jaw) to the implants were used. At baseline, the deepest site around each tooth or implant was recorded and used for later analysis. This deepest probing pocket depth of each unit (implant or tooth) was sampled for 30 s with sterile paper points at three different time points (VDW, 29 mm, ISO 15, Taper.04, Munich, Germany; Fig. 1D). In vivo sample collection was carried out under inhalation anesthesia with isofluran (2.5–5 vol%, Piramal GmbH, Hallbergmoos, Germany). At each session, the samples were taken from the same unit side. According to a previously published study, no buffer was used for the bacterial samples and afterwards the samples were stored in tubes (Eppendorf tubes, 1.5 ml, VWR International GmbH, Langenfeld, Germany) at − 80 °C [11].

In the laboratory, paper point samples were rehydrated by incubation in sterile 200 µl 0.95% sodium chloride (NaCl) solution (B. Braun Melsungen AG, Melsungen, Germany) for 30 min. After vortexing for 30 s, a dilution of 10^–1^ was prepared in sterile 0.95% saline solution and subsequently plated on Columbia blood agar plates (CBA) and on yeast-cysteine blood agar plates (HCB). The CBA agar plates were incubated at 37 °C and 5–10% CO_2_ atmosphere for 5 days to cultivate aerobic and facultative anaerobic bacteria. The HCB agar plates were incubated at 37 °C for 10 days (anaerobic chamber, GENbox; bioMérieux, Marcy l’Etoile, France) to cultivate anaerobic bacteria. The colony-forming units (CFU) on the agar plates were counted and the number of CFU per ml of the original sample was calculated. After Gram stains and determination of the cell morphology (Axioscope; Zeiss, Jena, Germany; 1000 × magnification), the pure bacterial isolates were determined using MALDI-TOF MS analysis as described in detail elsewhere [19]. In brief, a MALDI Biotyper Microflex LT (Bruker Daltonik, Bremen, Germany) was used according to the manufacturer’s recommendations. After sample preparation, mass spectra of the different pure bacterial colonies were acquired and compared with a reference database (Biotyper 3.0 software; Bruker Daltonik). The comparison resulted in different scores that identified the isolates at species level (score ≥ 2.0) or at genus level (score ≤ 2.0).

### Scanning electron microscopy (SEM), energy-dispersive X-ray spectroscopy (EDX), and transmission electron microscopy (TEM) analysis

Directly after sacrifice of the animals at the follow-up that took place at 3 months, the SEM, EDX, and TEM analyses were conducted for each group (either test- or control groups) of the peri-implant bone of the upper rat jaw. The region of interest for sample collection was defined as the area around the implant of 3 mm. For each method nine collected samples were used. The samples for SEM were fixed in 3% glutaraldehyde in 0.1 M Sorensen’s phosphate buffer, dehydrated in an ascending ethanol series (30–100%), and dried at 37 °C (SEM and EDX) according to a previously published study [33]. The samples were analyzed using an environmental scanning electron microscope (ESEM XL 30 FEG; FEI, Eindhoven, Netherlands) in backscatter mode with an acceleration voltage of 15 kV. EDX analysis was performed with the EDAX Genesis system (EDAX, Mahwah, NJ, United States). EDX analyses were performed at 8 random measurement points on each sample image, using a mean value for statistical analysis. EDX analysis measured elements of bone composition, such as Carbon, Oxygen, Natrium, Phosphate, Sulfur and Calcium.

For TEM, the samples were fixed in 3% glutaraldehyde in 0.1 M Sorensen’s phosphate buffer and decalcified in EDTA. After post-fixation in 1% OsO4 (Roth, Karlsruhe, Germany) in a 17% sucrose buffer, the samples were dehydrated in an ascending ethanol series, incubated in propylene oxide (Serva, Heidelberg, Germany), and embedded in Epon resin (Serva). Ultrathin Sects. (70–100 nm) were cut and stained with 0.5% uranyl acetate and 1% lead citrate (both EMS, Munich, Germany) to enhance contrast. Samples were viewed at an acceleration voltage of 60 kV using a Zeiss Leo 906 TEM (Carl Zeiss, Oberkochen, Germany). The TEM images were evaluated in the context of a descriptive investigation, with the aim of analyzing the condition of the bone cells. Specifically, the authors looked for different cell types, their structure, states of the cell organelle, cell membrane, cell nucleus and possible abnormalities.

### Statistical analysis

The sample size was calculated using the nQuery Advisor software (Version 8; Statsols, Cork, Ireland), with McNemar’s test on the equality of paired samples. Using a 0.05 significance level, an odds ratio of 0.15 [31], and power of 80% [34], a group comparison of the target main study parameter produced a sample size of N = 18 rats per group, including two drop-outs.

Analyses were performed using the Prism 8 software for Mac OS X (GraphPad; La Jolla, CA, USA) running on Apple OS X. Variables were analyzed using the Kolmogorov–Smirnov normality test. Kruskal–Wallis and Dunn’s multiple comparison tests were used to identify the inter- and intragroup differences in the total bacterial count. EDX analyses were compared using the Mann–Whitney test. A *p* value of < 0.05 was considered statistically significant.

## Results

Two animals from Group 2 were lost, one during anesthesia in the course of the operation, probably owing to respiratory arrest, and the second during medication in the rat restrainer. Therefore, out of 54 animals, 52 rats could be included in this evaluation.

**Fig. 2:**
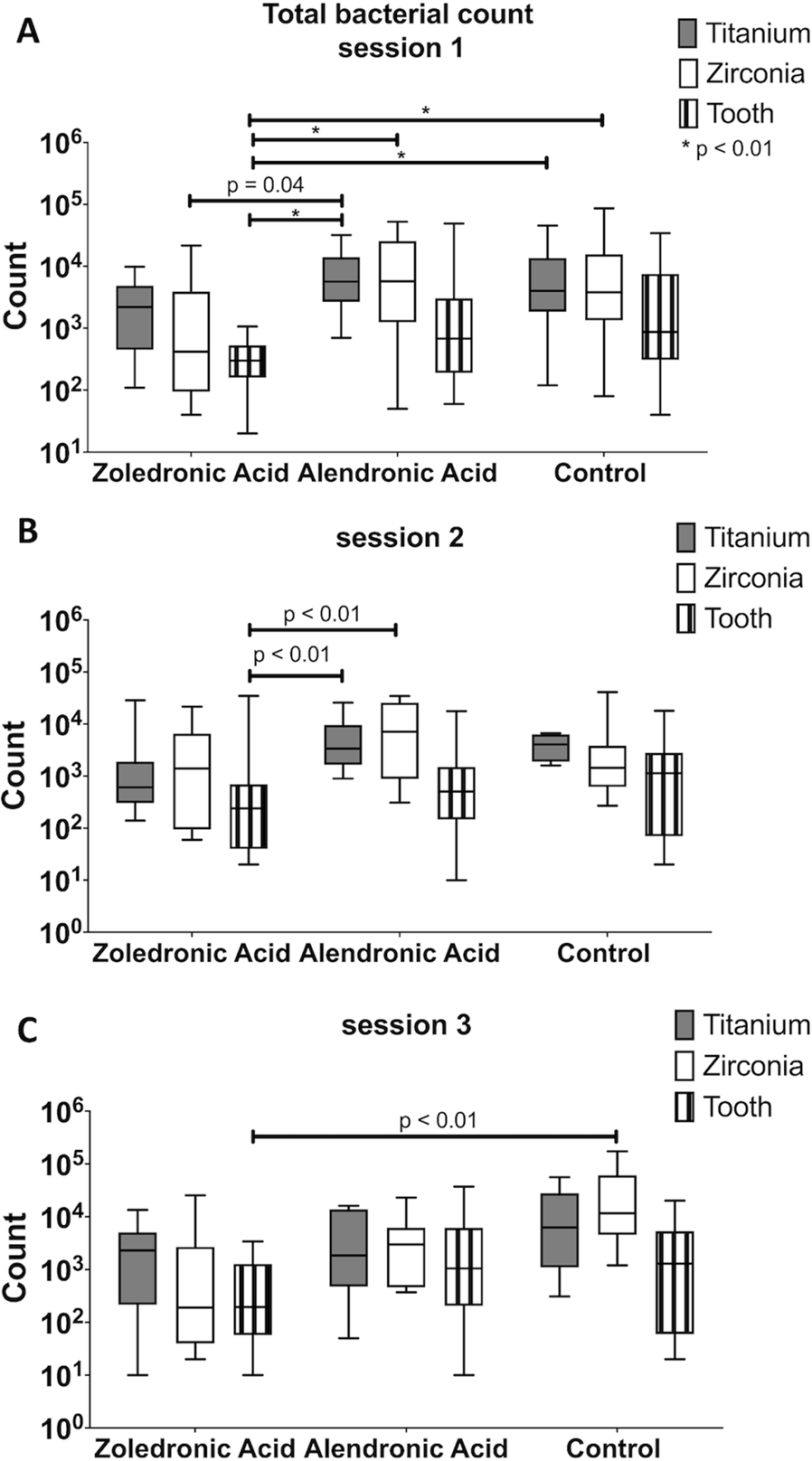
Total bacterial count measurements were taken at timepoints 1 week after surgery in session 1 (**A**), after 8 weeks in session 2 (**B**), and after 12 weeks in session 3 (**C**)

Regarding the total bacterial count, several significant differences were found in session 1 (after 1 week) between the groups (Fig. 2). Natural teeth in rats with zoledronic acid application showed a significantly lower adherent bacterial count when compared to the titanium and zirconia implants of the other groups (*p* < 0.01). Furthermore, titanium implants in the alendronic acid group (Group 2) exhibited a higher bacterial count when compared to the zirconia implants in the zoledronic acid group (Group 1; *p* = 0.04). The data of the probing depths of session 1 showed the lowest depths around the natural teeth (tooth mean 1.09 mm), regardless of whether the control- or the test groups were investigated. When evaluating the groups without medication, pocket values of zirconia- and titanium implants were significantly larger when compared to natural teeth (Zr control mean: 1.82 mm, Ti control mean: 1.81 mm; *p* > 0.01). When comparing the implant materials with bisphosphonate medication, no significant inter- and intragroup differences were found (Zr of group 1 mean: 1.33 mm, Ti of group 1 mean: 1.44 mm, Zr of group 2 mean: 1.56 mm, Ti of group 2 mean: 1.83 mm; *p* < 0.05).

In session 2 (after 8 weeks), significant differences regarding the bacterial count were noticeable between the natural teeth and both implant materials of Group 2 (alendronic acid). Less significant differences were recorded after 3 months, at which time only the zirconia material of the control group, Group 3, showed a higher bacterial count than the teeth of Group 1 (*p* < 0.01).

Additionally, intra-group changes over time in the zirconia implants revealed a significant value increase between sessions 1 (after 8 weeks) and 3 (after 12 weeks) (*p* = 0.01) and sessions 2 and 3 (*p* < 0.01, Table 1).

**Table 1:** Descriptive and statistical values for total bacterial count measurements between intragroup sessions (SD = standard deviation)

Tukey's multiple comparisons test	Titanium implant	Zirconia implant	Tooth
*Zoledronic acid*			
Mean	2.73E+03	3.83E+03	1.17E+03
SD	4.54E+02	6.81E+02	1.12E+03
Session 1 versus Session 2	0.99	0.99	0.74
Session 1 versus Session 3	0.95	0.99	0.99
Session 2 versus Session 3	0.99	1.00	0.83
*Alendronic acid*			
Mean	6.96E+03	9.73E+03	4.15E+03
SD	1.77E+03	4.75E+03	1.86E+03
Session 1 versus Session 2	0.81	0.99	0.57
Session 1 versus Session 3	0.60	0.50	0.93
Session 2 versus Session 3	0.93	0.61	0.35
*Control*			
Mean	9.41E+03	1.76E+04	4.02E+03
SD	5.36E+03	1.60E+04	1.52E+03
Session 1 versus Session 2	0.55	0.71	0.49
Session 1 versus Session 3	0.41	0.01	0.89
Session 2 versus Session 3	0.16	< 0.01	0.79

A* p* value of < 0.05 was considered statistically significant

**Fig. 3:**
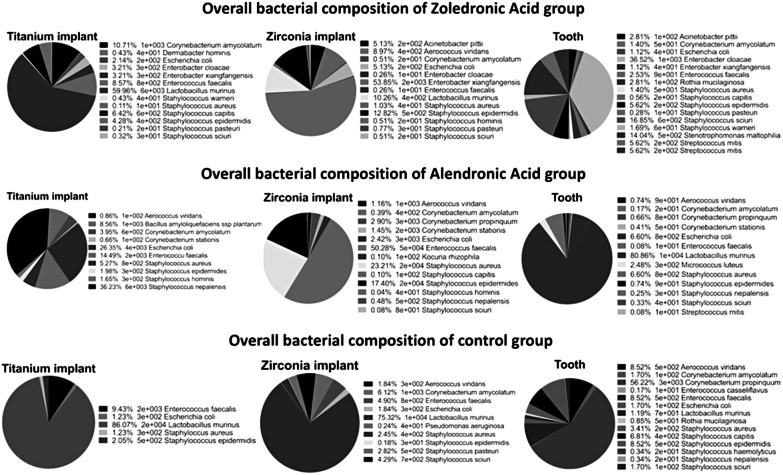
Overall bacterial composition for all groups (zoledronic acid, alendronic acid, and control group)

As seen in Fig. 3, overall bacterial composition showed that *Lactobacillus murinus* was the most predominant bacterium in several subgroups (Group 1 titanium implant, Group 2 tooth, Group 3 both implant materials). In addition, numerous other bacteria of the digestive tract of rats were detected around the tested implants and teeth (e.g., *Escherichia coli*, *Enterobacter cloacae*, and *Enterococcus fecalis*).

**Fig. 4:**
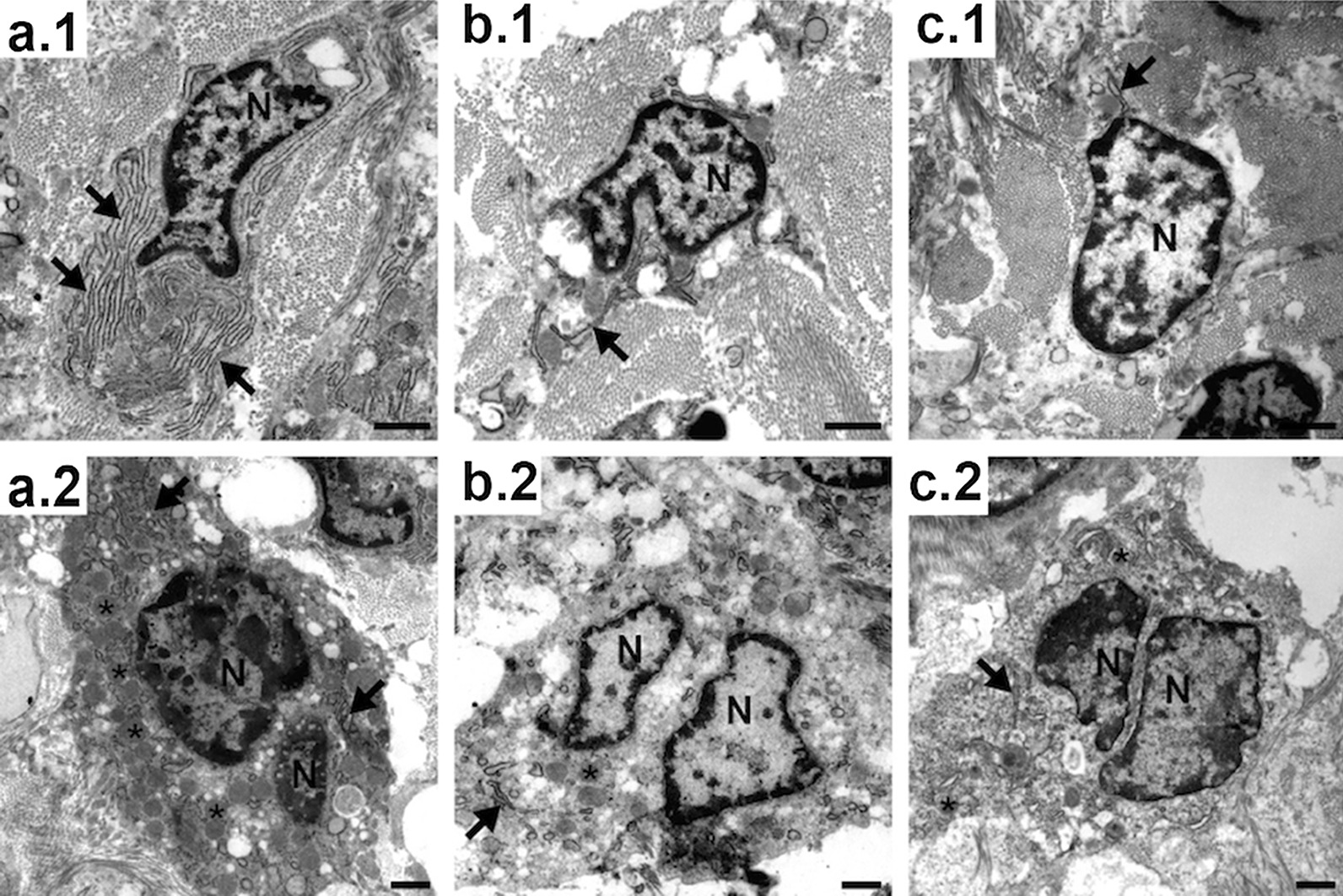
TEM pictures of bone cells from control animals (**a**) in comparison to zoledronic acid-treated (**b**) and alendronic acid-treated (**c**) animals. **a.1**–**c.1** show osteoblasts in the chondrogenic zone. **a.2**–**c.2** show osteoclasts. *Arrows: endoplasmic reticulum; asterisks: vesicles; N: nucleus

**Fig. 5:**
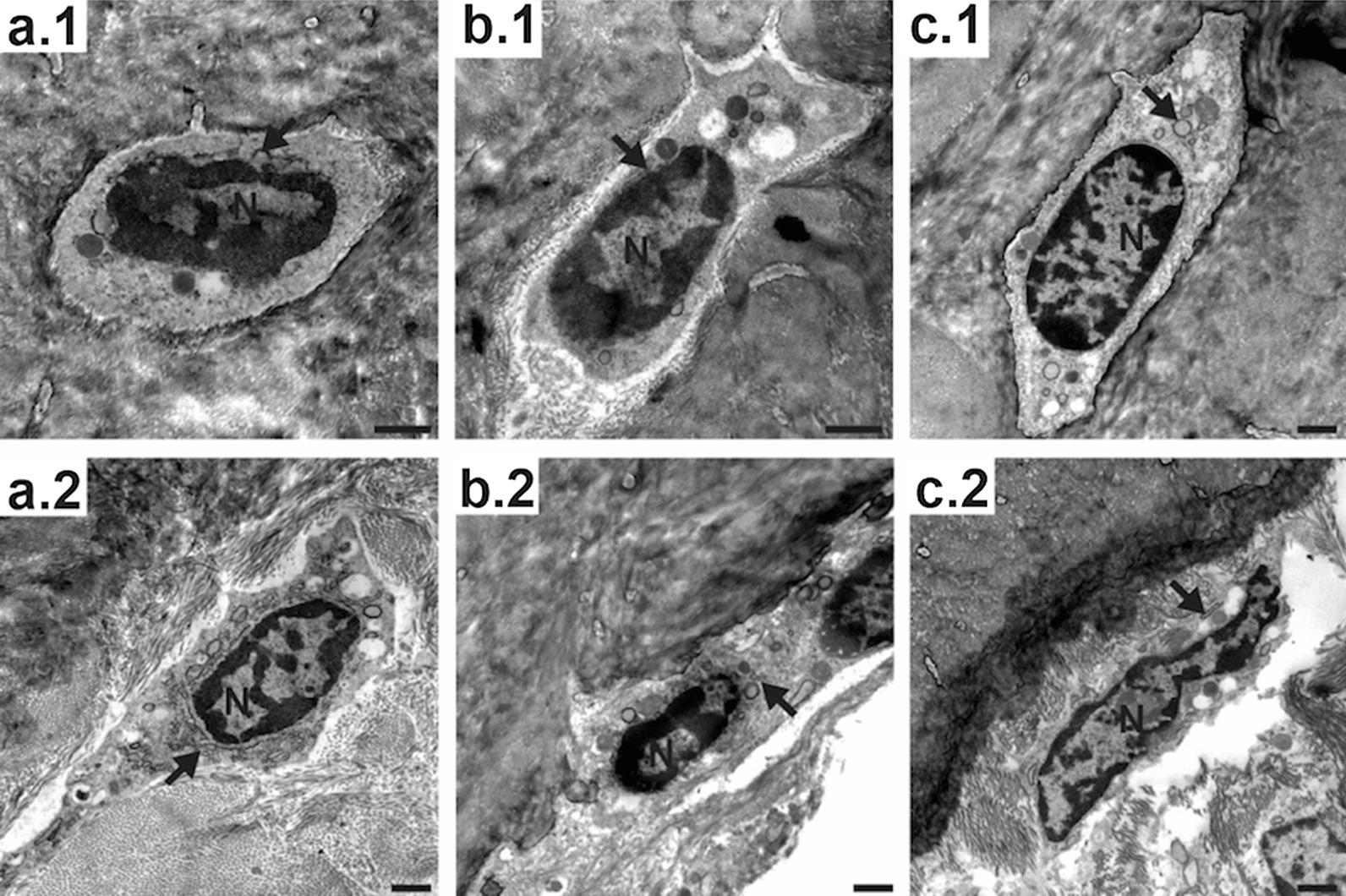
TEM pictures of osteocytes within the mineralized bone (**a.1**–**c.1**) and epithelioid osteoblasts on the surface of the mineralized bone of control animals (**a.2**–**c.2**), zoledronic acid-treated (**b**), and alendronic acid-treated (**c**) animals. *Arrows: endoplasmic reticulum; asterisks: vesicles; N: nucleus

In the untreated control group (Group 3), TEM analysis showed that the osteoblasts in the chondrogenic zone (Fig. 4a.1) had a strongly developed endoplasmic reticulum. This indicated a high protein synthesis rate and therefore high bone formation activity. In contrast, the endoplasmic reticulum of the osteoblasts in drug-treated animals (Fig. 4b.1 with zoledronic acid and Fig. 4c.1 with alendronic acid) was significantly less developed, indicating less activity. This effect was not seen in the osteoblasts at the mineralized bone edge (Fig. 5a–c.1) or the osteocytes within the ossified bone (Fig. 5a–c.2).

The number of osteoclasts in the samples of the treated animals (Fig. 5b.2, c.2) was higher than in the untreated control group (Fig. 5a.2), but most of these osteoclasts had only few Golgi structures and endoplasmic reticula, as well as fewer vesicles loaded with enzymes for dissolving bone material. The cytoplasm was pale, in contrast to the cytoplasm of active osteoclasts, in which high mitochondrial activity turns the cytoplasm acidophilic, resulting in dark contrast staining in electron microscopy. This and the low abundance of organelles suggested a less active or less developed state of these osteoclasts.

As shown in Table 2, the EDX analyses revealed that the calcium weight of the maxillary rat bone was significantly higher in the alendronic acid group when compared to the control group (*p* = 0.03). The zoledronic acid group also showed higher values but without any statistical significance. On the other hand, the control group without medication exhibited a higher carbon value in relation to the test groups with a significance of *p* = 0.04 to the zoledronic acid group. This is because the proportion of calcium increases. The ratio of calcium (Ca) to carbon (C) and phosphate (P) was formed to normalize the calcium measurements to the organic component density. The ratio of Ca to P was higher in both groups with bisphosphonate medication, as well as the ratio of Ca to C.

**Table 2:** Descriptive and statistical values for EDX analysis of bone composition between groups

Element	Control (n = 9)				Zoledronic acid (n = 9)				Alendronic acid (n = 9)				*p* value
	Mean	SD	Min	Max	Mean	SD	Min	Max	Mean	SD	Min	Max	
*Weight%*													
Carbon	47.37	31.63	12.82	86.09	22.94	3.87	19.87	31.20	25.93	5.08	21.28	34.51	*Con-Zol p* = *0.04*
Oxygen	24.94	14.89	5.40	47.34	38.14	13.65	21.86	55.21	29.66	9.64	10.35	43.66	
Natrium	2.11	0.65	1.21	3.12	2.00	0.33	1.42	2.46	1.67	0.36	1.25	2.39	
Phosphate	10.72	6.59	1.26	17.10	14.64	3.34	9.90	19.68	14.89	3.66	9.02	21.18	
Sulfur	0.59	0.91	0.00	2.24	0.00	0.00	0.00	0.00	0.00	0.00	0.00	0.00	
Calcium	14.28	12.01	0.26	29.62	22.28	11.43	10.73	37.82	27.86	10.48	12.94	45.74	*Con-Ale p* = *0.03*
*Ratio*													
Ca/C	0.30	0.38	0.02	0.34	0.97	2.96	0.54	1.21	1.07	2.06	0.61		
Ca/P	1.33	1.82	0.21	1.73	1.52	3.43	1.08	1.92	1.87	2.86	1.43		

SD = standard deviation, min = minimum and max = maximum

## Discussion

The purpose of this study was to assess the effect of the implant material under systemic bisphosphonate medication on the peri-implant composition and development of bacterial deposits. A clinical study in humans showed that the soft tissues around titanium implants developed a stronger inflammatory response to experimental plaque accumulation than those around zirconium implants and natural teeth in terms of total bacterial cell number [11]. The process of inflammation around the teeth and implants is very complex and not understood in depth; nevertheless, the total bacterial count can be seen as an overall indication of inflammation level [11]. Regarding the total bacterial count in our study, no significant inter-material differences were found at 3-month follow-up. Thus, the oral sulcus fluid of the rats affected the materials equally and there were no differences due to the different surface compositions. At session 1 the lowest bacterial count was evaluated around natural teeth, which also had the lowest probing depths. The probing depth might have an influence on bacterial count, as deeper pockets might be associated with different bacterial composition than shallow pockets. When interpreting the bacterial count, the probing depth should be considered as well. Pocket depths of implant materials with bisphosphonate medication showed no significant inter- and intragroup differences, however, titanium implants in the alendronic acid group (Group 2) exhibited a higher bacterial count when compared to the zirconia implants in the zoledronic acid group.

Although it is recognized that the use of rodent models to study human oral microbiota is debatable and may not provide an accurate representation [35], several common microbial species share the oral cavities of humans and rodents [36]. One study investigated the bacterial profile and bone healing in rats receiving doses of bisphosphonates [37]. Oral lesions were colonized mainly by non-pathogenic bacteria such as *Staphylococcus pasteuri*, *Streptococcus parasanguinis*, and *Streptococcus mitis*. In this study, numerous other bacteria of the digestive tract of rats were detected in the oral cavity. The explanation could be that rats eat their excrement from time to time. Rats break down food in the intestine that is rich in raw fiber and absorb a part of the nutrients thus gained through their own feces. We found that *L. murinus* was the most predominant bacterium around teeth/implants, followed by bacteria such as *E. coli*, *E. cloacae*, and *E. fecalis*. An animal model of mixed bacterial infection by oral lavage with human bacteria has been previously published [38]. A limitation of this bacterial analysis was that no statement about the viability of the bacteria was possible. Therefore, we decided to investigate the predominant microbial ecology of rats, focusing on quantitative data. Nevertheless, additional molecular DNA-based methods should be used also to characterize the adherent microbiota, since the culture technique does not detect the majority of bacteria in their natural niches [39].

Bisphosphonates are a class of drugs initially designed to prevent excessive osteoclast activity from causing bone loss [40]. TEM analysis in the present study showed that the low abundance of organelles hinted at a less active or less developed state of the osteoclasts of the samples that were treated with bisphosphonates. This effect was not seen in the osteoblasts at the mineralized bone edge or the osteocytes within the ossified bone, which may be because the osteoblastic cells in these locations are less active in general. With regard to the reduced activity of osteoclasts in our test groups, the results were in agreement with the literature. Bisphosphonates show signs of lower osteoclast activity [41, 42]. Furthermore, medication-related osteonecrosis of the jaw secondary to bisphosphonate therapy specimens had considerably more osteoclasts in terms of quantity, diameter, and nuclearity than the control specimens [43]. In another study alendronate treatment was also associated with an increase in the number of osteoclasts [44].

A similar study found that the administration of bisphosphonates to Sprague–Dawley rats improved Ca and P levels [45]. In bisphosphonates, the phosphorus atom is directly bonded to the carbon atom (not via an oxygen atom as in organic phosphate). While phosphates can easily be split off enzymatically by phosphatases, this is not the case for the P–C–P bonds; the bisphosphonates are therefore very stable in the body. The Ca/P concentration ratio increases, whereas the C/Ca ratio decreases in the healing bone matrix. The Ca/C ratio provides information on the degree of calcification of the bone matrix at points [46] of the respective measure, whereas the Ca/P ratio is positively related to induced bone loss [47]. The ratio of Ca to P was higher in both groups with bisphosphonate medication, as well as the ratio of Ca to C. The increased calcification of the test groups of our study indicated that the administered medication was effective.

A limitation of this study was that microbial ecology can vary between our rat model and humans.

## Conclusion

Systemic bisphosphonate delivery led to a traceable effect on the bone composition and the cells around the implants and teeth. Regarding microbiological parameters, neither implant material was superior to the other at 3-month follow-up. Based on our results, if patients with a previous history of bisphosphonates are to be treated with implants, the bacterial accumulation properties of the implant material should not be a decisive factor in the choice of material.

## Data Availability

The datasets used and/or analysed during the current study are available from the corresponding author on reasonable request.
